# Reaching a consensus on research priorities for supporting women with autoimmune rheumatic diseases during pre-conception, pregnancy and early parenting: A Nominal Group Technique exercise with lay and professional stakeholders

**DOI:** 10.12688/wellcomeopenres.14658.1

**Published:** 2018-06-20

**Authors:** Rhiannon Phillips, Denitza Williams, Daniel Bowen, Delyth Morris, Aimee Grant, Bethan Pell, Julia Sanders, Ann Taylor, Ernest Choy, Adrian Edwards

**Affiliations:** 1 Division of Population Medicine, Cardiff University, Cardiff, UK; 2 University Library Service, Cardiff University, Cardiff, UK; 3 Centre for Trials Research, Cardiff University, Cardiff, UK; 4 School of Healthcare Sciences, Cardiff University, Cardiff, UK; 5 Centre for Medical Education, Cardiff University, Cardiff, UK; 6 Division of Infection and Immunity, Cardiff University, Cardiff, UK

**Keywords:** Autoimmune rheumatic diseases, arthritis, Lupus, vasculitis, pregnancy, family planning, parenting, research priorities, consensus, Nominal Group Technique

## Abstract

**Background:**Women with autoimmune rheumatic diseases (ARDs) find it difficult to get information and support with family planning, pregnancy, and early parenting. A systematic approach to prioritising research is required to accelerate development and evaluation of interventions to meet the complex needs of this population.

**Methods:**A Nominal Group Technique (NGT) exercise was carried out with lay and professional stakeholders (n=29). Stakeholders were prepared for debate through presentation of available evidence. Stakeholders completed three tasks to develop, individually rank, and reach consensus on research priorities: Task 1 – mapping challenges and services using visual timelines; Task 2 - identifying research topics; Task 3 - individually ranking research topics in priority order. Results of the ranking exercise were fed back to the group for comment.

**Results:**The main themes emerging from Task 1 were the need for provision of information, multi-disciplinary care, and social and peer support. In Task 2, 15 research topics and 58 sub-topics were identified around addressing the challenges and gaps in care identified during Task 1.  In Task 3, a consensus was reached on the ten research topics that should be given the highest priority. These were individually ranked, resulting in the following order of priorities (from 1 – highest to 10 – lowest): 1. Shared decision-making early in the care pathway; 2. Pre-conception counseling; 3. Information about medication use during pregnancy/breastfeeding; 4. Personalised care planning; 5. Support for partners/family members; 6. Information about local support/disease specific issues; 7. Shared decision-making across the care pathway; 8. Peer-support; 9. Social inequalities in care, and; 10. Guidance on holistic/alternative therapies.

**Conclusions:**This systematic approach to identification of research priorities from a multi-disciplinary and lay perspective indicated that activities should focus on development and evaluation of interventions that increase patient involvement in clinical decision-making, multi-disciplinary models of care, and timely provision of information.

## Introduction

Women affected by autoimmune rheumatic diseases (ARDs), such as inflammatory arthritis, systemic lupus erythematous and vasculitis, during their childbearing years can face a range of challenges as these diseases and some treatments for these diseases may affect fertility, contraceptive choices, pregnancy outcomes, and breastfeeding ^1– 3^. Around a third of women with rheumatoid arthritis who are taking medication that is contraindicated in pregnancy, such as methotrexate and leflunomide, use ineffective or no contraception ^4, 5^. Women with ARDs are less likely to have children, have fewer children, and have longer intervals between pregnancies than healthy women, which are influenced by maternal choice, being advised to limit family size, altered sexual functioning, differences in fertility, and pregnancy loss ^6– 8^. The impact of rheumatic diseases on physical functioning can also impact on the daily activities associated with parenting ^9^. More integrated care and better information and counselling around pregnancy and early parenting for women with ARD and other chronic diseases have been recommended ^10– 15^.

A systematic review highlighted that there is little high quality research on pre-conception counselling for women with chronic health conditions ^12^. Similarly, a systematic review of interventions to improve knowledge and self-management skills around contraception, pregnancy and breastfeeding in women with rheumatoid arthritis ^11^ identified only one study that specifically evaluated education or self-management focused on pregnancy ^16^. This was a Randomised Controlled Trial (RCT) with 142 women in Australia, which found that a decision aid for women with RA to support their decision making about starting a family or having more children improved their knowledge about rheumatoid arthritis and pregnancy, and reduced decisional conflict compared with the control group ^16^.

European League Against Rheumatism (EULAR) guidelines have recently been produced providing recommendations on the management of family planning, assisted reproduction, pregnancy and menopause in systemic lupus erythematosus or antiphospholipid syndrome using a Delphi method ^17^. The EULAR guidelines advocate provision of information on family planning as early as possible following diagnosis, and provide guidance on the medical management of disease and reproductive health in women with these diseases and risk stratification ^17^. EULAR and British Society of Rheumatology/British Health Professionals in Rheumatology guidelines have been produced with regard to the use of anti-rheumatic and analgesic medication during pregnancy ^18– 20^.

In an Australian Delphi study, a panel of rheumatologists, obstetricians/obstetric physicians, and pharmacists was convened to reach consensus on key educational messages and clinical practice behaviour with regard to providing a consistent approach to care for women with rheumatoid arthritis in the areas of general health, contraception, conception and pregnancy, breastfeeding, and early parenting ^21^. A consensus was reached that guiding principles were that information delivery should be: coordinated; delivered in an appropriate mode and format, at the right time, and tailored to the individual patient; based on best available evidence; delivered by the right health professionals at the right time, and; adopt a non-judgmental approach to infant feeding ^21^.

Mixed-methods studies in Australia ^15^ and in the United Kingdom ^22^ indicate that women find it difficult to access consistent and high quality information on the use of medication during pregnancy planning, pregnancy and early parenting ^22^. Women with ARDs interact with a range of health and social care services during the period when they are thinking about or are building a family, including rheumatology, obstetrics, fertility clinics, midwifery and community nursing (health visiting), physiotherapy, and psychology/counselling services ^22^. Women and health professionals recognise the importance of well-coordinated multi-disciplinary care to meet the complex needs of this population ^15, 21, 22^.

The current study investigated what the priorities for research are in the United Kingdom, where the healthcare system differs to Australia in the way services are structured and commissioned ^23^. This built upon previous consensus studies in this field ^17, 21^ by capturing the views of patients and a range of professionals, including those who deliver community-based as well as secondary care services. We sought to consult with these stakeholders to reach a consensus on the areas of uncertainty that most require investigation to guide clinicians and researchers working in this field.

## Methods

**Figure 1.**
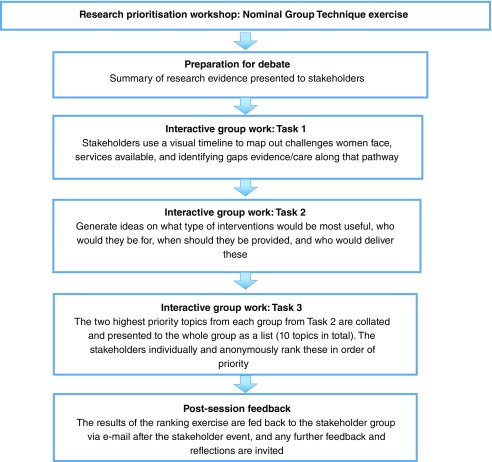
Overview of the Nominal Group Technique process for reaching a consensus on research priorities.

A Nominal Group Technique (NGT) exercise was carried out that included patients, researchers, and health professionals from a range of disciplines. The NGT ^24^ is a commonly used consensus method in medical and health service research, which uses small group discussions to provide prompt results for researchers ^25^. NGTs are highly structured and involve generation and sharing of ideas, clarification of ideas, and voting, with several variations of the technique having been reported in published literature ^25– 28^. An overview of the structure of the NGT consensus exercise used in our stakeholder workshop is provided in Figure 1. The NGT was part of a larger mixed-methods project; ‘Starting a family when you have an autoimmune rheumatic disease’ - the STAR Family Study. The STAR Family Study also included an online survey (n=128) and qualitative interviews with women (n=22) and health professionals (n=7), the findings of which are reported in full elsewhere ^22^.

### Participants

A stakeholder event was held in Cardiff (United Kingdom) in January 2017 to discuss the support provided to women with ARDs in relation to family planning, pregnancy and early parenting, and to reach a consensus on research priorities. The event was advertised via the project social media feeds (Twitter and Facebook; @STARfamilystudy) and website ( www.starfamilystudy.yolasite.com), and the Eventbrite website. In addition to the public adverts, health professionals, researchers and patient representatives in the research team’s institutions and professional networks were purposively sampled to ensure that a range of views was captured from patients, health professionals working in community, family practice, and hospital-based services, researchers, government, and voluntary sector organisations. Registration for the event was free. Travel and accommodation expenses were paid for patient representatives and students who attended the event. Patient representatives were paid for their time in line with Involving People guidance (£75 per half day of involvement) ^29^.

### Procedure

The first step in the NGT exercise was to prepare the participating stakeholders for debate. To provide the context for the NGT exercise, presentations were given on the lived experiences of women in the UK who were considering pregnancy, pregnant, or had young children (<5 years of age) based on the preliminary findings of a mixed-methods study that included an online survey (n=128) and qualitative interviews (n=22) with women who had ARDs (findings reported in full elsewhere) ^22^. The group was presented with the findings of a rapid literature review carried out by the research team prior to the event that set out to identify and assess the quality of evidence from RCTs of non-pharmacological interventions aiming to improve health and well-being outcomes for women with ARDs and their children, which focused on pre-conception, pregnancy and/or early parenting. Supplementary File 1 contains the rapid review protocol (Section A), study selection flow diagram (Section B), included studies (Section C), Cochrane assessment of bias (Section D) and studies excluded at full-text screen (Section E). Only three manuscripts (from two studies) ^16, 30, 31^ were identified that met the inclusion criteria for the rapid review. This built upon an earlier review by Ackerman *et al.* ^11^, with both reviews indicating that there is a significant gap in the evidence relating to how best to support this population. Brief presentations were given to provide the context for the NGT exercise and to stimulate discussion on: gender and pain; drugs and breastfeeding, and; shared decision-making in clinical settings. A summary of the event and the presentation slides are available at http://starfamilystudy.yolasite.com/event.php.

Stakeholders were asked to form six groups around the tables. A member of the research team facilitated each group. Each of the lay members joined a different group so that they and the professional stakeholders could share their views and experiences during discussions. Three group work tasks were used during the NGT exercise.

**Figure 2.**
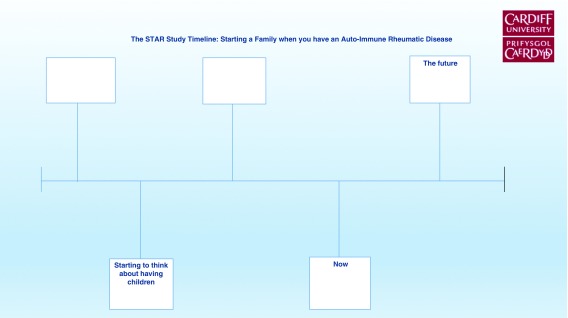
Visual timeline template used as an example for stakeholder during Task 1.

In the Task 1, the groups used visual timelines to map out women’s journeys toward starting a family, identifying challenges, where different services were provided, and to identify gaps in care and support along the pathway. Large sheets of paper, coloured marker pens, images relating to conception, pregnancy, early parenting and managing long-term conditions were provided, along with various items of stationary so that the groups could map out women’s journey towards building a family. Figure 2 shows the example timeline template used during the task. Stakeholders could use the template if they wanted to, but were free to present their ideas visually in whichever way they felt best represented their ideas. A break was provided before moving on to the next group work activity so that participants could look at the timelines created by the other groups.

In Task 2, participants reflected on the discussions and timelines created during Task 1 to generate ideas on how the challenges women faced and the gaps in care might be addressed. Based on this, they generated a list of research topics. Each group was asked to discuss and agree which of the two research topics they had identified during Task 2 were the highest priority topics. These were fed back to the larger group.

In Task 3, the 10 highest priority research topics were agreed by stakeholders, and these topics were individually ranked by the participants in order of priority (1-high to 10-low). The rank assigned to topics could be based on ‘quick wins’, most urgent need, and/or the interventions likely to have the highest impact based on the individual’s judgment. Ranking was completed anonymously on paper and collected by the research team at the end of the session. Findings of the individual ranking exercise were fed back to the stakeholder group via e-mail after the event to provide an opportunity for further feedback and reflection.

## Results

The NGT exercise was attended by 29 people, including four patients with ARDs, two General Practitioners (one of whom had specialism in rheumatology), a consultant in pain medicine, two midwives, a pharmacist, two physiotherapists, a health visitor, an occupational therapist, a clinical psychologist, ten researchers, three students, and a government representative.

During Task 1, stakeholders mapped out the challenges women faced on their journey towards building a family, the services that were available, and gaps in care/the evidence base. The stages most discussed by stakeholders were pre-conception, pregnancy, birth, and the post-natal period. Over-arching themes emerged in relation to the need for information (particularly about safety of medication and potential risks associated with starting/enlarging a family), multi-disciplinary care, and support from family and peers. Stakeholders felt that women could fall through the gaps in busy services, and that having clear care pathways and guidelines, a key worker, and ensuring that the quality of communication was good could be helpful in preventing this.

Specific challenges were highlighted at different stages of women’s journey towards building a family. At the pre-conception stage, stakeholders noted that conversations about contraception should be framed within an open discussion with women their preferences and options with regard to planning a family. The information provided needed to be realistic, high quality, and evidence based. The need to involve partners in discussions was emphasised.

During pregnancy, a need for information about medication was identified, which posed a challenge for health professionals due to a lack of available evidence on the safety of some drugs during pregnancy and breastfeeding. At this stage, information about birth options was needed. This was recognised as a source of anxiety for women and it was acknowledged that birth experiences could affect mental health post-natally. Planning for birth and the post-natal period during pregnancy was considered to be important.

During the post-natal period, needs shifted towards more practical and community-based support, such as midwives and health-visiting services, support with childcare, and social support. The antenatal period was considered by patients to be a time when their needs were not well met, as antenatal wards and maternity services were not equipped to manage their complex needs. They felt that training and raising awareness amongst health professionals working with families would be helpful in addressing this. A need for support with infant feeding was identified, and in particular advice about expressing milk for babies born prematurely and managing the effects of abrupt cessation of breastfeeding if women became unwell and/or needed to resume medication. Pain and fatigue were particular challenges that were identified at the early parenting stage. Ability to access health and community-based services was a challenge for women who were caring for young children. Later in the parenting stage of women’s journeys, meeting the needs of children and partners when women were unwell was considered important, in particular supporting their mental health and ensuring support was in place with transporting children to and from school and with childcare if or when this was required.

In Task 2, the groups built upon the discussions that took place during Task 1 to identify research topics, focusing on the type of interventions that could be used to improve the information and care provided to women with ARDs. The 15 topics and 58 sub-topics identified during Task 2 are shown in Table 1. Table 2 shows the 10 research topics that were considered to have the highest priority, in the order in which they were ranked during Task 3 (individual ranking exercise).

**Table 1.** Research topics and sub-topics emerging from Task 2.

Topics (presented in alphabetical order)	Sub-topics
Alternative therapies	Professional guidance on what is safe and effective, holistic approaches to care
Care pathways	Personalised care planning, core support worker, ‘prudent’ healthcare, tailored support packages, accessibility of services, ‘one stop shop’ approach
Co-production	Research led by women’s experiences
Information provision	Need to provide information early on, mode of delivery (written, leaflets, use of visual materials, videos, Skype and apps, demonstrations), pre-conception counselling, high quality, locally relevant information on support/ resources/links, ensuring relevant information is provided with prescriptions for medications, provision of practical advice (tips, products/aids available)
Clinical guidelines	Best practice, care pathways, applicable to all health professionals who work with families (e.g. midwives and health visitors)
Equipment	Hiring/loaning equipment to support women with pregnancy/early parenting, need for adaptation of tools/aid and innovation to meet the needs of parents
Multi-disciplinary care	Community level care, counsellors, primary and secondary care physicians, occupational therapy, appropriate referrals
Pain management	Alternatives to medical approaches, e.g. input from physiotherapists
Peer-support	Access to experiences of others & information, healthcare professional facilitation, support for peer-supporters, online peer-support, accessing peer-support, evidence underpinning peer-support approaches
Psychological interventions	Timely access, urgency, accessibility, use of apps/helplines, cost issues, Cognitive Behaviour Therapy, counselling/talking therapies
Safety of medication	Need to build the evidence base and provide accurate information through the whole journey from pre- conception to parenting
Shared decision-making	Mode of delivery (who, where, when, how?), patient activation, building skills and knowledge, shared decision- making as a long-term process not a one-off event, use of decisions aids/decision support tools
Social inequalities in health	Socioeconomic status, access to healthcare, gender, ethnic diversity, cultural differences
Support networks	Support from relatives, involving partners in decision making, ‘safe spaces’ for partners to explore issues, signposting to support groups, interventions that advocate partners’ involvement
Support/training for health professionals	‘Spotting the signs’ that women need additional support, knowledge of appropriate services

**Table 2.** Ranking of research topics in priority order by the lay and professional stakeholder group.

Ranking Highest (1) to lowest (10) priority	Topic	Summary of comments
1	Early shared decision- making (SDM)	Research on incorporating SDM early on in the care pathway was advocated, with an emphasis on the need to equip women as well as health professionals with the skills needed to engage in SDM.
2	Pre-conception care	Pre-conception was identified as a critical time during which good quality timely discussion on starting a family needs to happen.
3	Information: medication	High quality, evidence-based, consistent information needs to be provided to women on the use of disease modifying and analgesic medication during pregnancy and breastfeeding.
4	Personalised care	Individual women’s needs and the availability of local services vary, and pathways of care can be unclear. Ways of providing more personalised care should be investigated, e.g. dedicated case-workers to develop needs-based individually tailored packages of care.
5	Support for women’s social network	The group highlighted the need to investigate the support needs of women’s close social network, such as partners and family members, and for the women themselves. This could include counselling and social support for partners and family members.
6	Information: specific to disease and local area	The need for research on information needs in relation to specific diseases and tailored to the local area (due to variability in services), e.g. using leaflets and educational materials about local services and support, and condition/treatment specific information to guide women through their journey to motherhood.
7	SDM across the care pathway	As well as incorporating SDM early on after diagnosis, research was required about how SDM can be incorporated at every stage of women’s journeys towards parenthood, including particularly pain control. Ideas for supporting consistent use of SDM included a 'one stop shop' with counsellors, primary care physicians, and other members of the multi-disciplinary team in developing long-term care plans.
8	Peer-support	Research on peer-support was considered important, as this is widely used by women but the quality and impact of this type of support is untested. Research on high quality, online, peer-support was advocated, including how health professionals might interact with this and how it could be tailored to local health service contexts.
9	Health inequalities	The group expressed concerns about growing inequalities in health, and how this might impact on women with autoimmune rheumatic diseases. A need for research on tackling social inequalities in health, and developing equity-enhancing interventions was highlighted.
10	Holistic/alternative therapies	The need for evidence on safety and efficacy of alternative and holistic therapies (taking into account potential placebo effects), and providing professional guidance to women on their use was also important.

One additional reflection was received following the e-mail invitation for post-workshop reflections on the rankings: peer-support and better evidence/information about complementary and alternative therapies were given a relatively low research rank in the NGT individual ranking exercise, whereas many women in the previous mixed-methods study had expressed a need for this kind of support ^22^.

## Discussion

The NGT exercise highlighted the broad range of areas where the quality of information and care that women with ARDs receive during pre-conception, pregnancy and early parenting could be improved. The lay and professional stakeholder group reached a consensus that research focusing on improving shared decision-making in healthcare, high quality conversations during the pre-conception stage, evidence-based information on medication use during pregnancy and breastfeeding, and more personalised approaches to care had a high priority. Our stakeholder group also acknowledged the importance of generating evidence relating to peer-support and alternative therapies, as these were areas of support that were highly valued by women but where there is a lack of high quality evidence relating to their mechanisms, safety, and efficacy.

A previous Delphi study with clinicians carried out in Australia ^21^ highlighted the importance of providing consistent information to women with Rheumatoid Arthritis across the whole journey from pre-conception through to early parenting, and of adopting a whole-person approach. Themes that arose related to health promotion and prevention of disease, disease management, guidance on obtaining reliable and trustworthy information, and discussion of family planning that involves partners ^21^. Information on family planning early on after diagnosis has also been identified as a priority for women with Systemic Lupus Erythematous and antiphospholipid syndrome using a modified Delphi method with an expert group ^17^. Several of these themes overlapped with the research topics identified in the current study, particularly with regard to the need for high quality information early on after diagnosis ^17, 21^, involvement of partners, and adoption of a holistic approach ^21^. Shared-decision making and care planning were identified as high priority areas for research in the current study, which is consistent with the prominence of patient-centred approaches to care in UK health policy ^32– 35^. Peer-support and the role of partners were also prominent themes in our NGT discussions, whereas these were only touched upon in the Delphi studies with secondary care clinicians ^30^.

In the rapid review that was carried out to inform the NGT discussions, we found very little high quality evidence that could guide clinical practice in meeting the complex information and support needs of women of reproductive age who have an autoimmune rheumatic disease. Two studies (three published manuscripts) were identified that were eligible for inclusion in the rapid review ^16, 30, 31^. The Meade *et al.* ^16^ study investigated the use of a ‘motherhood choices’ decision aid for women with rheumatoid arthritis, with women who received the decision aid showing improved knowledge about pregnancy and arthritis and reduced decisional conflict. The Cravioto ^30^ and Sanchez-Guerrero ^31^ manuscripts reported on the safety, acceptability, and side-effects of a progesterone only pill, combined oral contraceptive, and a copper intra-uterine device, along with counselling and specialized health attention, for women with systemic lupus erythematous. They found that disease activity was mild and stable across the three intervention groups for the duration of the trial, and there were no between-group differences in disease activity or flares ^31^. Side-effects not related to their disease were also similar across the intervention groups, but the progesterone-only pill had lower acceptability ^30^. This scarcity of evidence relating to provision of information and support for women with long-term limiting illnesses, including rheumatoid arthritis, was consistent with the findings of previous relevant systematic reviews that have highlighted a research gap in this area ^11, 12^.

### Implications for practice

Numerous clinical reviews, guidelines, and observational studies have highlighted the need for provision of information on family planning early on following diagnosis, pre-conception counselling, co-ordination/joined-up care, and multi-disciplinary team involvement for women of reproductive age who have an autoimmune rheumatic disease [e.g. 1, 5, 6, 10, 17, 36– 48. There is little evidence to guide us on what the most effective, cost-effective, and acceptable interventions are to better meet the complex needs of this population. The current study builds upon previous consensus studies with expert groups ^17, 21^, contributing to a growing body of evidence that a high priority should be given to improving information and developing patient-centred holistic models of care for women with ARDs during family planning, pregnancy, and early parenting.

The implementation of best practice and dissemination of the latest evidence can be challenging ^49^. As well as providing high quality evidence on the optimal approaches to disease management and the safety and efficacy of medication, research in this area should investigate designing healthcare systems that incorporate well-coordinated multi-disciplinary, and patient centred approaches. Interventions that draw upon community-based resources, including women’s own social networks and peer-support should also be investigated. Researchers, clinicians, and funding bodies need to prioritise research in this area to strengthen the evidence base, and improve outcomes for women of reproductive age who have an ARD and their children.

### Strengths and weaknesses of the study

The strengths of this study were that an established and systematic consensus methodology was used, taking a broad multi-disciplinary and lay perspective, to prioritise topics for research in this highly under-researched area. The NGT exercise was useful in gathering the views of a range of stakeholders, including patients and multi-disciplinary group of healthcare professionals working in community, primary, and secondary care settings, and reaching a consensus on research topics. The composition of the group involved in an NGT is recognized as a limitation of the approach, as this can affect the generalisability of the findings ^50^. Rheumatology nurses and obstetric consultants were not present at the workshop, and priorities may have altered had they been present. Further, healthcare systems vary widely between countries in the way that they are structured and funded, and the extent to which these findings would transfer to other cultures and health systems would require further investigation.

## Conclusions

This systematic approach to the development of research priorities with women with ARDs and a multi-disciplinary group of health professionals indicates that activities should focus on the development and evaluation of interventions that increase patient involvement in clinical decision-making, multi-disciplinary models of care, and timely provision of high quality information. Given the scarcity of high quality research in this area, an increased awareness of research priorities should guide researchers and health and social care professionals in focusing their activities.

## Ethical approval

Ethical approval for the STAR Family Study, within which this work was embedded, was granted by the Cardiff University School of Medicine Research Ethics Committee on 20/10/16 (reference number 16/56). Stakeholders were asked for permission verbally to publish a summary of the discussions, but written consent was not required as this was a stakeholder engagement activity and no personal information was collected from participants.

## Data availability

All data underlying the results are available as part of the article and no additional source data are required.

## Supplementary materials

Supplementary File 1: Rapid review information. Section A, rapid review protocol; Section B, Rapid review flow diagram of selection of manuscripts; Section C, Table i: Included studies; Section D, Table ii: Quality assessment; Section E, Table iii: Studies excluded at full text stage.

## References

[1] Østensen M Cetin I: Autoimmune connective tissue diseases. *Best Pract Res Clin Obstet Gynaecol.* 2015;29(5):658–670. 10.1016/j.bpobgyn.2015.03.003 25891380

[2] Signore C Spong CY Krotoski D: Pregnancy in Women With Physical Disabilities. *Obstet Gynecol.* 2011;117(4):935–947. 10.1097/AOG.0b013e3182118d59 21422868

[3] Soh MC Nelson-Piercy C: High-risk pregnancy and the rheumatologist. *Rheumatology (Oxford).* 2015;54(4):572–587. 10.1093/rheumatology/keu394 25477056

[4] Østensen M von Esebeck M Villiger PM: Therapy with immunosuppressive drugs and biological agents and use of contraception in patients with rheumatic disease. *J Rheumatol.* 2007;34(6):1266–1269. 17516615

[5] Clowse ME: Managing contraception and pregnancy in the rheumatologic diseases. *Best Pract Res Clin Rheumatol.* 2010;24(3):373–385. 10.1016/j.berh.2009.12.004 20534371

[6] Olesińska M Ostanek L Majdan M: Fertility, pregnancy planning, and pharmacotherapy during the pregnancy, postpartum and breastfeeding period in patients with rheumatoid arthritis and other inflammatory arthropathies. *Reumatologia/Rheumatology.* 2014;52(1):7–21. 10.5114/reum.2014.41446

[7] Katz PP: Childbearing decisions and family size among women with rheumatoid arthritis. *Arthritis Rheum.* 2006;55(2):217–223. 10.1002/art.21859 16583405

[8] Østensen M: New insights into sexual functioning and fertility in rheumatic diseases. *Best Pract Res Clin Rheumatol.* 2004;18(2):219–232. 10.1016/j.berh.2004.01.002 15121041

[9] Katz PP Pasch LA Wong B: Development of an instrument to measure disability in parenting activity among women with rheumatoid arthritis. *Arthritis Rheum.* 2003;48(4):935–943. 10.1002/art.10990 12687535

[10] Doria A Bajocchi G Tonon M: Pre-pregnancy counselling of patients with vasculitis. *Rheumatology (Oxford).* 2008;47(Suppl 3):13–15. 10.1093/rheumatology/ken152 18504277

[11] Ackerman IN Ngian GS Van Doornum S: A systematic review of interventions to improve knowledge and self-management skills concerning contraception, pregnancy and breastfeeding in people with rheumatoid arthritis. *Clin Rheumatol.* 2016;35(1):33–41. 10.1007/s10067-015-3131-7 26638162

[12] Steel A Lucke J Adams J: The prevalence and nature of the use of preconception services by women with chronic health conditions: an integrative review. *Bmc Womens Health.* 2015;15:14. 10.1186/s12905-015-0165-6 25783639 PMC4338627

[13] Ngian GS Briggs AM Ackerman IN: Safety of anti-rheumatic drugs for rheumatoid arthritis in pregnancy and lactation. *Int J Rheum Dis.*Online First.2016;19(9):834–43. 10.1111/1756-185X.12860 27125255

[14] Ostensen M: Connective tissue diseases: Contraception counseling in SLE--an often forgotten duty? *Nat Rev Rheumatol.* 2011;7(6):315–316. 10.1038/nrrheum.2011.54 21519353

[15] Ackerman IN Jordan JE Van Doornum S: Understanding the information needs of women with rheumatoid arthritis concerning pregnancy, post-natal care and early parenting: A mixed-methods study. *BMC Musculoskelet Disord.* 2015;16:194. 10.1186/s12891-015-0657-4 26285693 PMC4545539

[16] Meade T Dowswell E Manolios N: The motherhood choices decision aid for women with rheumatoid arthritis increases knowledge and reduces decisional conflict: a randomized controlled trial. *BMC Musculoskelet Disord.* 2015;16:260. 10.1186/s12891-015-0713-0 26395873 PMC4579637

[17] Andreoli L Bertsias GK Agmon-Levin N: EULAR recommendations for women's health and the management of family planning, assisted reproduction, pregnancy and menopause in patients with systemic lupus erythematosus and/or antiphospholipid syndrome. *Ann Rheum Dis.* 2017;76(3):476–485. 10.1136/annrheumdis-2016-209770 27457513 PMC5446003

[18] Götestam Skorpen C Hoeltzenbein M Tincani A: The EULAR points to consider for use of antirheumatic drugs before pregnancy, and during pregnancy and lactation. *Ann Rheum Dis.* 2016;75(5):795–810. 10.1136/annrheumdis-2015-208840 26888948

[19] Flint J Panchal S Hurrell A: BSR and BHPR guideline on prescribing drugs in pregnancy and breastfeeding-Part I: standard and biologic disease modifying anti-rheumatic drugs and corticosteroids. *Rheumatology (Oxford).* 2016;55(9):1693–1697. 10.1093/rheumatology/kev404 26750124

[20] Flint J Panchal S Hurrell A: BSR and BHPR guideline on prescribing drugs in pregnancy and breastfeeding-Part II: analgesics and other drugs used in rheumatology practice. *Rheumatology (Oxford).* 2016;55(9):1698–1702. 10.1093/rheumatology/kev405 26750125

[21] Briggs AM Jordan JE Ackerman IN: Establishing cross-discipline consensus on contraception, pregnancy and breast feeding-related educational messages and clinical practices to support women with rheumatoid arthritis: an Australian Delphi study. *BMJ Open.* 2016;6(9):e012139. 10.1136/bmjopen-2016-012139 27633637 PMC5030591

[22] Phillips R Pell B Grant A: Identifying the unmet information and support needs of women with autoimmune rheumatic diseases during pregnancy planning, pregnancy and early parenting: mixed-methods study.In Press. 10.1186/s41927-018-0029-4 PMC6390539 30886972

[23] Policies EOoHSa: Health Systems in Transition summary: Australia.In.: World Health Organisation (Europe);2006 Reference Source

[24] Delbecq AL Van de Ven AH: A group process model for problem identification and program planning. *J Appl Behav Sci.* 1971;7(4):466–492. 10.1177/002188637100700404

[25] McMillan SS King M Tully MP: How to use the nominal group and Delphi techniques. *Int J Clin Pharm.* 2016;38(3):655–662. 10.1007/s11096-016-0257-x 26846316 PMC4909789

[26] Hiligsmann M van Durme C Geusens P: Nominal group technique to select attributes for discrete choice experiments: an example for drug treatment choice in osteoporosis. *Patient Prefer Adherence.* 2013;7:133–139. 10.2147/PPA.S38408 23412964 PMC3572758

[27] Allen J Dyas J Jones M: Building consensus in health care: a guide to using the nominal group technique. *Br J Community Nurs.* 2004;9(3):110–114. 10.12968/bjcn.2004.9.3.12432 15028996

[28] Vella K Goldfrad C Rowan K: Use of consensus development to establish national research priorities in critical care. *BMJ.* 2000;320(7240):976–980. 10.1136/bmj.320.7240.976 10753149 PMC27337

[29] National Institute for Health Research: Budgeting for involvement: Practical advice on budgeting for activity involving the public in research studies.2013; (accessed 8/5/18). Reference Source

[30] Cravioto MD Jiménez-Santana L Mayorga J: Side effects unrelated to disease activity and acceptability of highly effective contraceptive methods in women with systemic lupus erythematosus: a randomized, clinical trial. *Contraception.* 2014;90(2):147–153. 10.1016/j.contraception.2014.04.001 24815101

[31] Sánchez-Guerrero J Uribe AG Jiménez-Santana L: A trial of contraceptive methods in women with systemic lupus erythematosus. *N Engl J Med.* 2005;353(24):2539–2549. 10.1056/NEJMoa050817 16354890

[32] Welsh Government: Well-being of Future Generations (Wales) Act 2015.2015 Reference Source

[33] Welsh Government: Prudent healthcare: Securing health and well-being for future generations.2016 Reference Source

[34] Care Quality Commission: Guidance for Providers on Meeting the Regulations: Health and Social Care Act 2008 (Regulated Activities) Regulations 2014.2015; (accessed 1.5.18). Reference Source

[35] Fund TKs: From vision to action: Making patient-centred care a reality.2012; (accessed 1/5/18). Reference Source

[36] Østensen M Andreoli L Brucato A: State of the art: Reproduction and pregnancy in rheumatic diseases. *Autoimmun Rev.* 2015;14(5):376–386. 10.1016/j.autrev.2014.12.011 25555818

[37] Ostensen M Brucato A Carp H: Pregnancy and reproduction in autoimmune rheumatic diseases. *Rheumatology (Oxford).* 2011;50(4):657–664. 10.1093/rheumatology/keq350 21097449

[38] Ateka-Barrutia O Khamashta MA: The challenge of pregnancy for patients with SLE. *Lupus.* 2013;22(12):1295–1308. 10.1177/0961203313504637 24098002

[39] Baer AN Witter FR Petri M: Lupus and Pregnancy. *Obstet Gynecol Surv.* 2011;66(10):639–653. 10.1097/OGX.0b013e318239e1ee 22112525

[40] Chakravarty E Clowse ME Pushparajah DS: Family planning and pregnancy issues for women with systemic inflammatory diseases: patient and physician perspectives. *BMJ Open.* 2014;4(2):e004081. 10.1136/bmjopen-2013-004081 24500612 PMC3918989

[41] Fischer-Betz R Späthling-Mestekemper S: [Pregnancy and inflammatory rheumatic diseases]. *Z Rheumatol.* 2013;72(7):669–82. 10.1007/s00393-013-1223-9 23989692

[42] Jain V Gordon C: Managing pregnancy in inflammatory rheumatological diseases. *Arthritis Res Ther.* 2011;13(1):206. 10.1186/ar3227 21371350 PMC3157639

[43] Märker-Hermann E Fischer-Betz R: Rheumatic diseases and pregnancy. *Curr Opin Obstet Gynecol.* 2010;22(6):458–465. 10.1097/GCO.0b013e3283404d67 20966752

[44] Moroni G Ponticelli C: Pregnancy in women with systemic lupus erythematosus (SLE). *Eur J Intern Med.* 2016;32:7–12. 10.1016/j.ejim.2016.04.005 27142327

[45] Neeman N Aronson MD Schulze JE: Improving pregnancy counseling for women with rheumatoid arthritis taking methotrexate. *Am J Med.* 2009;122(11):998–1000. 10.1016/j.amjmed.2009.07.009 19854323

[46] Ngian GS Briggs AM Ackerman IN: Management of pregnancy in women with rheumatoid arthritis. *Med J Aust.* 2016;204(2):62–63. 10.5694/mja15.00365 26821101

[47] Østensen M: Contraception and pregnancy counselling in rheumatoid arthritis. *Curr Opin Rheumatol.* 2014;26(3):302–307. 10.1097/BOR.0000000000000044 24663105

[48] Schwarz EB Manzi S: Risk of unintended pregnancy among women with systemic lupus erythematosus. *Arthritis Rheum.* 2008;59(6):863–866. 10.1002/art.23712 18512717

[49] Kitson AL Rycroft-Malone J Harvey G: Evaluating the successful implementation of evidence into practice using the PARiHS framework: theoretical and practical challenges. *Implement Sci.* 2008;3(1):1. 10.1186/1748-5908-3-1 18179688 PMC2235887

[50] Peña A Estrada CA Soniat D: Nominal group technique: a brainstorming tool for identifying areas to improve pain management in hospitalized patients. *J Hosp Med.* 2012;7(5):416–420. 10.1002/jhm.1900 22190453

