# Supplementary material for: Reaching a consensus on research priorities for supporting women with autoimmune rheumatic diseases during pre-conception, pregnancy and early parenting: A Nominal Group Technique exercise with lay and professional stakeholders
Source: Wellcome Open Res. 2018 Jun 20;3:75. [Version 1] doi: 10.12688/wellcomeopenres.14658.1 (PMC6092904; doi:10.12688/wellcomeopenres.14658.1)
Supplement: Supplementary file 1 [file wellcomeopenres-3-15960-s0000.tgz › d7d15d9c-f4a2-4def-86b5-220a33f7d815.zip!/Supplementary File 1/Section A.docx]

**Supplementary File 1: Section A**

**Protocol for a rapid review of Randomised Controlled Trials (RCTs) of non-pharmacological interventions focusing on family planning, pregnancy and early parenting for women with autoimmune rheumatic diseases**

Rapid reviews are a more streamlined approach to evidence synthesis than traditional systematic reviews, which can be time consuming and resource intensive [1]. The purpose of the rapid review was to provide an up-to-date synthesis of high quality evidence relating to the use of non-pharmacological interventions to improve health and quality of life outcomes for women with autoimmune rheumatic diseases during pre-conception, pregnancy and early parenting that would enable us to identify uncertainties with regard to the information and support that should be provided to this population. We sought to build on the evidence provided by previous systematic reviews, which focused on rheumatoid arthritis [2], but not other forms of rheumatic disease, or looked at pre-conception counseling only in a broader range of illnesses [3].

***Rapid review search strategy***

Four electronic databases were searched for relevant records: Web of Knowledge, Medline, the Cochrane Database of Clinical Trials, and SCOPUS. Search terms related to disease group, starting a family, intervention, and research design. Following from the Ackerman *et al.* [2] review, searches were restricted to articles published between 2005 and 2016 due to significant changes over the last decade in the way that rheumatic diseases are managed [4]. The search terms used focused on disease, context, intervention type, and study design, as shown in Box 1.

**Box 1:** Search terms used for the rapid review

| **Category** |  | **Search terms** |
| --- | --- | --- |
| Disease |  | Arthritis or Rheumatic or Vasculitis or Rheumatoid Arthritis or Psoriatic Arthritis or Lupus or SLE or Juvenile Idiopathic Arthritis or JIA or Spondyloarthritis or Systemic Sclerosis or Polyangiitis or Ankylosing Spondylitis or Wegener or Churg-Strauss or Sjogren |
|  | AND |  |
| Context |  | Family planning or Contracept* or Conception or Pregnant or Pregnancy or Fertility or Reproductive or Childbearing or Natal or Prenatal or Perinatal or Antenatal or Postnatal or Pre-natal or Peri-natal or Ante-natal or Post-natal or Breastfeeding or Breastfed or Lactation or Parenting or Motherhood or Early years |
|  | AND |  |
| Intervention |  | Education* or Self management or Self care or Health literacy or Peer support or Expert patient or Counseling or Counselling or Shared decision making or Decision aid or Option grid or Communication skills or Cognitive Behaviour Therapy or Cognitive Behavior Therapy or Cognitive Therapy or Behavior Therapy or Behaviour Therapy or CBT or Mindfulness or Meditation or Hypnosis or Physiotherapy or Exercise or Physical activity or Diet or Nutrition or Occupational or Manipulation or Acceptance and Commitment Therapy or ACT or Yoga or Pilates or Swimming or Acupuncture or Music or Physical Therapy or Case management or Personalized care planning or Personalised care planning |
|  | AND |  |
| Study design |  | Clinical trial or randomized or randomised or RCT or CCT or placebo or randomly or trial or groups |

Search terms used to identify RCTs were based on a strategy that was developed in MEDLINE [5], which has comparable sensitivity and specificity, but greater precision, than the more complex Cochrane Highly Sensitive Search Strategy for identifying randomized trials in MEDLINE [6]. The structure of the searches was modified for each database based on the search options available. As this was a rapid review, we restricted the searches in MEDLINE and Web of Knowledge to ‘topic’ or ‘title and abstract’ fields respectively to ensure that the searches were sufficiently focused, while searches applied to ‘all fields’ in the Cochrane Database of Clinical Trials and SCOPUS databases. The reference lists of each paper included in the review, and of review articles of pre-conception services for women with long-term limiting illnesses [3] and interventions to improve knowledge and self-management skills for women with rheumatoid arthritis in relation to contraception, pregnancy, and breastfeeding [2] were screened to identify any additional papers that met the inclusion criteria.

***Inclusion criteria***

- RCT design
- Published in English between 2005 and 2016
- Population includes patients with inflammatory arthritis or inflammatory connective tissue disease (including Rheumatoid Arthritis, Systemic Lupus Erythematosus, Juvenile Idiopathic Arthritis, Systemic Sclerosis, Ankylosing Spondylitis, Psoriatic Arthritis, Sjogren’s Syndrome, Antiphospholipid Syndrome, or Vasculitis – e.g. granulomatosis with polyangiitis or Wegener’s, eosinophilic granulomatosis with polyangiitis or Churg-Strauss syndrome, and microscopic polyangiitis)
- Population includes women of reproductive age (16 to 49 years)
- Study examines our population of interest: i.e. women who are using contraception, planning a family, pregnant, breastfeeding and/or have children under 5 years of age
- Includes a non-pharmacological intervention specifically aiming to improve the health and/or well-being of women during family planning, pregnancy planning, pregnancy, breastfeeding and/or caring for young children (under 5 years of age)
- Includes at least one outcome measure relating to maternal and/or child health or quality of life, and mediators of these outcomes (e.g. knowledge, attitudes, self-efficacy, self-management skills, decisional conflict, quality of communication with health care professionals and access to health care services) in women of reproductive age who have autoimmune rheumatic diseases

***Exclusion criteria***

- Reviews and editorials of existing studies
- Study design not a RCT (including single case studies and observational studies)
- Studies using retrospective data only
- Animal studies
- Studies of pharmacological interventions only
- Conference abstracts where full findings are not available
- Studies that have specific exclusion criteria for women from the groups of interest (i.e. women who are planning a pregnancy, pregnant, breastfeeding or have young children, or women of reproductive age).

***Study selection and data extraction***

Two reviewers independently screened articles at the tile/abstract and full text stages to confirm eligibility for inclusion in the review (DB & RG at title/abstract, DB & RP at full text). Any disagreement was resolved by discussion. If two or more papers from the same dataset met the inclusion criteria for the review, each of these would be included but their relationship noted. Data were extracted from the full text papers by a single reviewer (RP) using a critical appraisal form based on CONSORT reporting standards for clinical trials [7], and extracted data were validated by a second member of the research team (DW). A systematic search strategy was used, and findings were reported in line with the PRISMA checklist [8]. To enable the rapid review to be completed with the time and resources available for this study, the review focused on high quality evidence and only included studies using an RCT design. Data were extracted from the studies included in the review relating to the markers of quality in reporting of clinical trials included the CONSORT statement. The Cochrane Risk of Bias tool [9] was used to assess quality of the included studies. Finally, a narrative synthesis of the included studies was produced [10].

**Rapid Review Protocol References**

1.	Khangura S, Konnyu K, Cushman R, Grimshaw J, Moher D: Evidence summaries: the evolution of a rapid review approach. *Systematic Reviews* 2012, 1(1):10.

2.	Ackerman IN, Ngian G-S, Van Doornum S, Briggs AM: A systematic review of interventions to improve knowledge and self-management skills concerning contraception, pregnancy and breastfeeding in people with rheumatoid arthritis. *Clinical rheumatology* 2016, 35(1):33-41.

3.	Steel A, Lucke J, Adams J: The prevalence and nature of the use of preconception services by women with chronic health conditions: an integrative review. *Bmc Womens Health* 2015, 15.

4.	Hyrich KL, Verstappen SMM: Biologic therapies and pregnancy: the story so far. *Rheumatology* 2014, 53(8):1377-1385.

5.	Glanville JM, Lefebvre C, Miles JNV, Camosso-Stefinovic J: How to identify randomized controlled trials in MEDLINE: ten years on. *Journal of the Medical Library Association* 2006, 94(2):130-136.

6.	Glanville JM, Lefebvre C, Miles JNV, Camosso-Stefinovic J: How to identify randomized controlled trials in MEDLINE: then years on. . *Journal of the Medical Library Association* 2006, 94(2):130-136.

7.	Schulz KF, Altman DG, Moher D: CONSORT 2010 Statement: updated guidelines for reporting parallel group randomised trials. *BMJ* 2010, 340.

8.	Liberati A, Altman DG, Tetzlaff J, Mulrow C, Gøtzsche PC, Ioannidis JPA, Clarke M, Devereaux PJ, Kleijnen J, Moher D: The PRISMA statement for reporting systematic reviews and meta-analyses of studies that evaluate healthcare interventions: explanation and elaboration. *BMJ* 2009, 339.

9.	Higgins JP, Altman DG, Gøtzsche PC, Jüni P, Moher D, Oxman AD, Savović J, Schulz KF, Weeks L, Sterne JA: The Cochrane Collaboration’s tool for assessing risk of bias in randomised trials. *Bmj* 2011, 343:d5928.

10.	Popay J, Roberts H, Sowden A, Petticrew M, Arai L, Rodgers M, Britten N, Roen K, Duffy S: Guidance on the conduct of narrative synthesis in systematic reviews. *A product from the ESRC methods programme Version* 2006, 1:b92.
