# Supplementary material for: Reaching a consensus on research priorities for supporting women with autoimmune rheumatic diseases during pre-conception, pregnancy and early parenting: A Nominal Group Technique exercise with lay and professional stakeholders
Source: Wellcome Open Res. 2018 Jun 20;3:75. [Version 1] doi: 10.12688/wellcomeopenres.14658.1 (PMC6092904; doi:10.12688/wellcomeopenres.14658.1)
Supplement: Supplementary file 1 [file wellcomeopenres-3-15960-s0000.tgz › d7d15d9c-f4a2-4def-86b5-220a33f7d815.zip!/Supplementary File 1/Section B.docx]

**Supplementary File 1: Section B**

**Figure 1:** Flow diagram for selection of papers for the rapid review
