# Supplementary material for: Reaching a consensus on research priorities for supporting women with autoimmune rheumatic diseases during pre-conception, pregnancy and early parenting: A Nominal Group Technique exercise with lay and professional stakeholders
Source: Wellcome Open Res. 2018 Jun 20;3:75. [Version 1] doi: 10.12688/wellcomeopenres.14658.1 (PMC6092904; doi:10.12688/wellcomeopenres.14658.1)
Supplement: Supplementary file 1 [file wellcomeopenres-3-15960-s0000.tgz › d7d15d9c-f4a2-4def-86b5-220a33f7d815.zip!/Supplementary File 1/Section C.docx]

**Supplementary File 1, Section C: Rapid Review Results Tables**

**Table i:** Characteristics of studies included in the rapid review

| **First author & year of publication** | **Setting & population** | **Inclusion/exclusion criteria** | **Intervention and control groups** | **Method of randomisation** | **Number randomized/analysed** | **Relevant outcomes** | **Key findings** |
| --- | --- | --- | --- | --- | --- | --- | --- |
| **Study 1:** Meade *et al* 2015^[1]^ | Community (recruited online), women with RA | Inclusion: Aged within childbearing/ rearing years, clinically diagnosed with RA and currently under the care of a rheumatologist, contemplating having children or more children  Exclusion: none | Intervention:  45 page motherhood decision aid resource (electronic and paper versions)  Control:  No intervention | Individually randomised, using Bernoulli function in Excel. | Randomised  N=188 (intervention n=107, control n=81)  Analysed  N=144 (intervention n= 78, control n=66) | Self-report questionnaires completed pre-and post intervention (between 4 and 12 weeks to complete both surveys):  Pregnancy in Rheumatoid Arthritis Questionnaire (PiRAQ), Decisional Conflict Scale (DCS), Arthritis Self-efficacy Scale (ASES), Hospital Anxiety and Depression Scale (HADS). | **PiRAQ**: Significant main effect of group, $\eta_{p}^{2}$= 0.128  Significant time by group interaction,$\eta_{p}^{2}$= 0.214  **DCS:** Significant time by group interaction, $\eta_{p}^{2}$= 0.117. No main effect of group.  **Secondary outcomes:** No difference between groups in HADS or ASES scores at follow-up. |
| **Study 2:** |  |  |  |  |  |  |  |
| Cravioto *et al* 2014^[2]^ | Hospital, Women with SLE | Inclusion: ≤40 years of age, willing to use a contraceptive method to prevent pregnancy  Exclusion: Severe lupus activity at baseline, vaginal bleeding of unknown etiology, blood pressure >140/90mmHg, liver or cardiovascular disease, platelet count <50,000 per cubric mm, history/presence of cancer or thrombosis, recent pelvic inflammatory disease, use of rifampicin or anticonvulsant medication, and heavy smoking in women >35 years old | Interventions:  Combined oral contraceptives (COCs)  Progestin-only pills (POPs)  Copper-releasing intrauterine devices (IUDs)  All groups received counseling from the study gynecologist | Individually randomised, using computer generated list.  Study co-ordinator, blind to contraceptive method, allocated participants to next available study number | Randomised  N=196  (COCs n=69, POPs n=57, IUDs n=70)  Analysed  N=162  (COCs n=54, POPs n=54, IUDs n=54) | Side effects unrelated to disease activity, blood pressure, weight, discontinuation rates for allocated contraceptive, unintended pregnancy rates | Significant differences (p<0.05) in discontinuation rates between groups for medical reasons (COC 11%, POP 31%, IUD 4%) and any reason (COC 35%, POP 55%, IUD 29%). 5 women had unintended pregnancies, with an estimated 1 year failure rate of 4.7 in the COC group, 5.4 in the POP group and 2.2 in the IUD group per 100 woman-years |
| Sànchez-Guerrero *et al* 2005^[3]^ |  | As above | As above | As Above | As above | General disease activity assessed using the Systemic Lupus Erythematosus Disease Activity Index (SLEDAI), maximum SLEDAI score, change in SLDEI score, incidence of lupus flares, median time to first flare, SLE treatment, adverse events | SLEDAI scores: no differences between intervention groups in global or maximum disease activity, incidence or probability of flares, or time to first flare. Adverse events: Thromboses occurred in four women (two from each of the hormonal contraceptive groups), severe infections occurred in 3 patients in the COC group, 2 in the POP group and 5 in the IUD group. One patient in the COC group died from amoxicillin-related severe neutropenia |

1.	Meade T, Dowswell E, Manolios N, Sharpe L: The motherhood choices decision aid for women with rheumatoid arthritis increases knowledge and reduces decisional conflict: a randomized controlled trial. *Bmc Musculoskeletal Disorders* 2015, 16.

2.	Cravioto MD, Jimenez-Santana L, Mayorga J, Seuc AH: Side effects unrelated to disease activity and acceptability of highly effective contraceptive methods in women with systemic lupus erythematosus: a randomized, clinical trial. *Contraception* 2014, 90(2):147-153.

3.	Sanchez-Guerrero J, Uribe AG, Jimenez-Santana L, Mestanza-Peralta M, Lara-Reyes P, Seuc AH, Cravioto MD: A trial of contraceptive methods in women with systemic lupus erythematosus. *The New England Journal of Medicine* 2005, 353(24):2539-2549.
