# Supplementary material for: Reaching a consensus on research priorities for supporting women with autoimmune rheumatic diseases during pre-conception, pregnancy and early parenting: A Nominal Group Technique exercise with lay and professional stakeholders
Source: Wellcome Open Res. 2018 Jun 20;3:75. [Version 1] doi: 10.12688/wellcomeopenres.14658.1 (PMC6092904; doi:10.12688/wellcomeopenres.14658.1)
Supplement: Supplementary file 1 [file wellcomeopenres-3-15960-s0000.tgz › d7d15d9c-f4a2-4def-86b5-220a33f7d815.zip!/Supplementary File 1/Section D.docx]

**Supplementary File 1: Section D**

**Table ii:** Quality assessment using Cochrane Risk of Bias tool for studies included in the rapid review

| **Bias** | **Study 1: Meade *et al.* 2015** | **Study 2: Cravioto *et al.* 2014 & Sànchez-Guerrero *et al.* 2005** |
| --- | --- | --- |
| Random sequence generation (selection bias) | Moderate risk: randomisation using the Bernoulli function in Excel, control | Unclear risk: computer-generated sequence, but no detail provided on method or software used to generate the sequence |
| Allocation concealment (selection bias) | High risk: participants randomized by researcher | Low risk: researcher allocated women to the next study number in the sequence, but did not know which trial arm that number had been allocated to |
| Blinding of participants and researchers (performance bias) | High risk: no blinding of participants or researchers | Moderate risk: Single blind design. Patients & gynecologist aware of trial arm allocation. Rheumatologist and researchers blind to trial arm |
| Blinding of outcome assessment (detection bias) | High risk: participants were followed up by researcher who was aware of allocation. Link to online survey for follow up questionnaire was sent to participants after the researcher had made follow up contact with them by phone or e-mail. | Low risk: Rheumatologist (assessing disease activity) and researchers blind to trial arm |
| Incomplete outcome data (attrition bias) | Moderate risk: 76% of women initially enrolled in the study completed both baseline and follow-up assessments. | Low risk: Data included in final analysis for 82.7% of patients who were randomised. |
| Selective reporting (reporting bias) | Low risk: assuming all measures included in the study are described in the methods, all relevant outcomes are reported on in the results. | Low risk: reported on main effects on disease activity, adverse events, side effects unrelated to disease activity and acceptability |
