# Supplementary material for: Reaching a consensus on research priorities for supporting women with autoimmune rheumatic diseases during pre-conception, pregnancy and early parenting: A Nominal Group Technique exercise with lay and professional stakeholders
Source: Wellcome Open Res. 2018 Jun 20;3:75. [Version 1] doi: 10.12688/wellcomeopenres.14658.1 (PMC6092904; doi:10.12688/wellcomeopenres.14658.1)
Supplement: Supplementary file 1 [file wellcomeopenres-3-15960-s0000.tgz › d7d15d9c-f4a2-4def-86b5-220a33f7d815.zip!/Supplementary File 1/Section E.docx]

**Supplementary File 1: Section E**

**Table iii:** Full text manuscripts excluded from the rapid review at the full-text stage

| **Lead author** | **Year of publication** | **Study Design** | **Population** | **Intervention** | **Primary reason for exclusion** |
| --- | --- | --- | --- | --- | --- |
| N. Brodin(1) | 2008 | RCT | Patients with early Rheumatoid Arthritis | Healthy physical activity coaching programme | Intervention does not focus on pregnancy planning, pregnancy or early parenting |
| G. McKellar(2) | 2007 | Non-randomised pilot study | Female patients with Rheumatoid Arthritis living in socially deprived areas of Glasgow | Mediterranean-type diet intervention | Not an RCT design, intervention does not focus on pregnancy planning, pregnancy or early parenting |
| S. de Brouwer(3) | 2011 | RCT | Patients with Rheumatoid Arthritis | Stress management training | Intervention does not focus on pregnancy planning, pregnancy or early parenting |
| V. Manning(4) | 2014 | RCT | People with Rheumatoid Arthritis of 5 years duration | Education, self-management and upper extremity exercise programme | Intervention does not focus on pregnancy planning, pregnancy or early parenting |
| T. Seneca(5) | 2015 | RCT | People with early Rheumatoid Arthritis | Partly supervised self-administered exercise programme | Intervention does not focus on pregnancy planning, pregnancy or early parenting |
| Y. el Miedany(6) | 2012 | RCT | Arthritis patients | Integration of Patient Related Outcome Measures with the implementation of a joint fitness programme | Intervention does not focus on pregnancy planning, pregnancy or early parenting |
| E. Tarakci (7) | 2013 | RCT | Patients with Juvenile Idiopathic Arthritis | Land-based home exercise programme | Intervention does not focus on pregnancy planning, pregnancy or early parenting |
| E. De Dios Sancho (8) | 2011 | RCT | Rheumatoid arthritis patients | Physical therapy treatment | Intervention does not focus on pregnancy planning, pregnancy or early parenting |

1.	Brodin N, Eurenius E, Jensen I, Nisell R, Opava CH, Grp PS. Coaching patients with early rheumatoid arthritis to healthy physical activity: A multicenter, randomized, controlled study. Arthritis & Rheumatism-Arthritis Care & Research. 2008;59(3):325-31.

2.	McKellar G, Morrison E, McEntegart A, Hampson R, Tierney A, Mackle G, et al. A pilot study of a Mediterranean-type diet intervention in female patients with rheumatoid arthritis living in areas of social deprivation in Glasgow. Annals of the Rheumatic Diseases. 2007;66(9):1239-43.

3.	de Brouwer SJM, Kraaimaat FW, Sweep FCGJ, Donders RT, Eijsbouts A, van Koulil S, et al. Psychophysiological Responses to Stress after Stress Management Training in Patients with Rheumatoid Arthritis. PLOS ONE. 2011;6(12):e27432.

4.	Manning VL, Hurley MV, Scott DL, Coker B, Choy E, Bearne LM. Education, Self-Management, and Upper Extremity Exercise Training in People With Rheumatoid Arthritis: A Randomized Controlled Trial. Arthritis Care & Research. 2014;66(2):217-27.

5.	Seneca T, Hauge EM, Maribo T. Comparable effect of partly supervised and self-administered exercise programme in early rheumatoid arthritis - a randomised, controlled trial. Danish Medical Journal. 2015;62(8).

6.	El Miedany Y, El Gaafary M, El Arousy N, Ahmed I, Youssef S, Palmer D. Arthritis education: the integration of patient-reported outcome measures and patient self-management. Clinical and Experimental Rheumatology. 2012;30(6):899-904.

7.	Tarakci E, Yeldan I, Baydogan SN, Olgar S, Kasapcopur O. EFFICACY OF A LAND-BASED HOME EXERCISE PROGRAMME FOR PATIENTS WITH JUVENILE IDIOPATHIC ARTHRITIS: A RANDOMIZED, CONTROLLED, SINGLE-BLIND STUDY. Journal of Rehabilitation Medicine. 2012;44(11):962-7.

8.	De Dios Sancho EP, Martin-Nogueras AM. [Influence of the physical therapy on the health and quality of life of the rheumatic patient]. Reumatologia clinica. 2011;7(4):224-9.
