# Supplementary material for: Reaching a consensus on research priorities for supporting women with autoimmune rheumatic diseases during pre-conception, pregnancy and early parenting: A Nominal Group Technique exercise with lay and professional stakeholders
Source: Wellcome Open Res. 2018 Jun 20;3:75. [Version 1] doi: 10.12688/wellcomeopenres.14658.1 (PMC6092904; doi:10.12688/wellcomeopenres.14658.1)
Supplement: Peer review history

# Peer review: Referee response for version 1

**DOI:** 10.21956/wellcomeopenres.15960.r33610

**Published:** 2018-08-14

**Authors:** Rebecca Fischer-Betz

**Affiliations:** 1 Policlinic of Rheumatology and Hiller Research Unit Rheumatology, Medical Faculty, Heinrich Heine University Düsseldorf (HHU), Düsseldorf, Germany

Women with autoimmune rheumatic diseases have clear family-planning-related educational needs and seek specific information and support to make decisions. However, information and service needs of women with rheumatic diseases concerning pregnancy, lactation and early parenting have been identified as being insufficient in recent studies.

 With this in mind, the authors of this article sought to reach a consensus on the most important areas of uncertainty that require investigation to guide clinicians and researchers working in this field.

 A structured method, the Nominal Group Technique was used as a process involving problem identification, solution generation, and decision making. Participants included lay and professional stakeholders from several disciplines. While the participation of patients must be commended, the workshops would probably have gained benefit from participants from other disciplines like obstetricians and teratology information specialists who both are frequently involved in the decision-making process in clinical life. In addition, the involvement of more physicians specialized in rheumatology with practical experience in the treatment of women with rheumatic diseases would have been desirable.

 Not unexpected but nevertheless astonishing is the result of the literature search with regard to high quality research on pre-conception counselling and care during pregnancy for women with ARDs. A similar experience has been reported in the recently published EULAR recommendations for women`s health and the management of family planning which were devised with the intention of helping physicians involved in the care of women with SLE and APS ^1^. Although various research groups have contributed significantly to improved knowledge of risk stratifications and optimal intervention before and during pregnancy, there is a lack of large, prospective studies and for several recommendations no high evidence could be found in the literature.

 Taken together, the paper of Phillips describes very well the current knowledge gap in relation to the state of research in patients with ARDs who consider family planning. The consensus on prioritizing research main themes rank the identified research topics and listed shared decision-making, pre-conception counseling, information about medication use during pregnancy/breastfeeding and personalized care planning over other points like guidance on holistic/alternative therapies. However, the authors make no recommendations as to what such research could look like. The noticeable lack of evidence in many areas may reflect the difficulty of conducting such studies. Within shared decision the physician should not only inform about the various options, but also explore the desired approach of the patient, recognise his fears, needs, expectations and wishes and his level of information and optimize the latter if necessary. Competence, gained through practice, may be a contributing factor to influence this process and at present, many rheumatologists do not feel sufficiently qualified for a complex situation like family planning ^2^. Behavioural studies are conceivable that aim to improve the communication skills between this particular group of patients and their health care providers. The cited study of Meade et al found that participants who were given the motherhood decision aid had a greater increase in knowledge around RA and pregnancy-related topics and a greater reduction in decisional conflict compared with a no-intervention control group ^3^. Research on how shared decision-making plays out in this group of patients could probably best be conducted at the already existing specialized family planning clinics in different countries. In addition, the development of a “standardized pregnancy counseling set” could also be done here. However, one also has to keep in mind that comparatively few women have access to these specialized centres, for example families living in rural areas or low- and middle-income countries. There is a special need for evidence-based and freely accessible information to support these women. The use of web-based therapeutic patient education or telemedicine in providing information and decision support could be subject of research on multi-disciplinary models of care.

 With regard to information on antirheumatic drug therapy, recommendations from the EULAR and the British Society for Rheumatology have recently been published which represent a useful aid to assist clinicians in counseling patients ^4^^-^^5^ on leaflets of anti-rheumatic drugs showed that at least half of them had information inconsistencies which may compromise the consensual decision-making process between patient and health care professional ^6^. This underlines the importance of dissemination and communication of clinical guidelines.

 There is a paucity of data regarding the safety of medications during pregnancy, mainly due to the ethical and logistical constraints. The challenge therefore is to get sufficient data on pregnancy exposures and appropriate controls. Registry-based studies are actually best fitted to get results but due to the rarity of exposures these studies are often time-consuming. Pregnancy registers for women with ARDs do exist in various countries and recently a European Network of Pregnancy Registers in Rheumatology was established which could provide the basis to collect large numbers of pregnancies and drug exposures prospectively.

 In summary, despite the prevalence of ARDs among women of childbearing age, it is clear that published models of disease education do not adequately cater to this important stage of life. The systematic approach by Phillips to identification research priorities points to the necessity of standardized strategies and could serve as a basis for future study design.

I have read this submission. I believe that I have an appropriate level of expertise to confirm that it is of an acceptable scientific standard.

## References

[1] AndreoliLBertsiasGKAgmon-LevinNBrownSCerveraRCostedoat-ChalumeauNDoriaAFischer-BetzRForgerFMoraes-FontesMFKhamashtaMKingJLojaconoAMarchioriFMeroniPLMoscaMMottaMOstensenMPamfilCRaioLSchneiderMSvenungssonETektonidouMYavuzSBoumpasDTincaniA: EULAR recommendations for women's health and the management of family planning, assisted reproduction, pregnancy and menopause in patients with systemic lupus erythematosus and/or antiphospholipid syndrome. *Ann Rheum Dis*.2017;76(3) : 10.1136/annrheumdis-2016-209770 476-485 10.1136/annrheumdis-2016-209770 27457513PMC5446003

[2] ChakravartyEClowseMEPushparajahDSMertensSGordonC: Family planning and pregnancy issues for women with systemic inflammatory diseases: patient and physician perspectives. *BMJ Open*.2014;4(2) : 10.1136/bmjopen-2013-004081 e004081 10.1136/bmjopen-2013-004081 24500612PMC3918989

[3] MeadeTDowswellEManoliosNSharpeL: The motherhood choices decision aid for women with rheumatoid arthritis increases knowledge and reduces decisional conflict: a randomized controlled trial. *BMC Musculoskelet Disord*.2015;16: 10.1186/s12891-015-0713-0 260 10.1186/s12891-015-0713-0 26395873PMC4579637

[4] Götestam SkorpenCHoeltzenbeinMTincaniAFischer-BetzRElefantEChambersCda SilvaJNelson-PiercyCCetinICostedoat-ChalumeauNDolhainRFörgerFKhamashtaMRuiz-IrastorzaGZinkAVencovskyJCutoloMCaeyersNZumbühlCØstensenM: The EULAR points to consider for use of antirheumatic drugs before pregnancy, and during pregnancy and lactation. *Ann Rheum Dis*.2016;75(5) : 10.1136/annrheumdis-2015-208840 795-810 10.1136/annrheumdis-2015-208840 26888948

[5] FlintJPanchalSHurrellAvan de VenneMGayedMSchreiberKArthanariSCunninghamJFlandersLMooreLCrossleyAPurushothamNDesaiAPiperMNisarMKhamashtaMWilliamsDGordonCGilesIBSR and BHPR Standards, Guidelines and Audit Working Group: BSR and BHPR guideline on prescribing drugs in pregnancy and breastfeeding-Part II: analgesics and other drugs used in rheumatology practice. *Rheumatology (Oxford)*.55(9) : 10.1093/rheumatology/kev405 1698-702 10.1093/rheumatology/kev405 26750125

[6] SabandoMOSaavedraMASequeiraGKerzbergE: Analysis of the data on pregnancy and lactation provided by patient information leaflets of anti-rheumatic drugs in Argentina. *Rheumatol Int*.2018;38(4) : 10.1007/s00296-018-3931-6 669-673 10.1007/s00296-018-3931-6 29332199

# Peer review: Referee response for version 1

**DOI:** 10.21956/wellcomeopenres.15960.r33477

**Published:** 2018-07-27

**Authors:** Monika Østensen

**Affiliations:** 1 High Risk Pregnancy Unit, Sorlandet Hospital, Kristiansand, Norway

Inspired by the difficulties of female patients with rheumatic disease to get information and support with family planning, pregnancy, and early parenting the authors analyzed research required to answer the unmet needs of this group.

 They used a Nominal Group Technique (NGT) to reach consensus on the most important research issues. NGT is a good method to gain group consensus on certain goals and where means to reach a goal must be prioritized. The technique creates lists of issues ranked as the most important for achieving a goal and issues that are less important to achieving the goal and are more difficult to perform.

 After a detailed preparation of background knowledge a group of different stakeholders was involved in the working groups. The selection of participants of the workshops would have benefitted from including obstetricians since they have an essential role during pregnancy, around delivery and early postpartum. Likewise, inclusion of nurses specialized in Rheumatology would have opened for more disease related input including quality of life.

 It is amazing that the search for literature revealed only two high quality studies that had investigated the effect of structured interventions in a systematic way. Specialized clinics caring for and managing women with rheumatic disease who plan a family or are pregnant have been in place at different University Hospitals in Europe and USA for several decades. Much of the data collected and published from these clinics supports main themes of research recommended in the present paper. However, the above-mentioned centres have not systematically investigated how the structured counselling they offer and the care they provide influences the female patients they serve. It is amazing that the effect of these interventions has not been studied in a randomized, controlled study though all experts agree that counselling and pre-conception and pregnancy care will improve pregnancy outcomes.

 The EULAR recommendations for women's health and the management of family planning in SLE and APS ^1^ describe a structured and practical approach, and in agreement with the present paper point to the necessity to support evidence.

 The paper of Phillips analyses many important needs of women on their way to motherhood which confirm previous studies performed in patients with rheumatic disease who consider family planning ^2^^,^^3^. However, the needs must be translated into research and it would be interesting to know what type of research the authors envision to be adequate. Which outcomes should be included: Pregnancy and child outcome, coping, quality of life, influence on health care costs?  Which groups should be compared: women/families receiving a certain intervention/service and those not receiving them? To identify needs is easier than to design research that could give clues on how to meet them and which interventions would make the greatest difference. The paper analyses the needs and ranks the most pressing ones but gives no hint how precisely each identified need should be translated into research that provides evidence that an intervention actually is useful.

 Some of the research issues of Table 2 are more straight forward than others. The safety and tolerability of drugs during pregnancy and lactation can be studied by prospective or register-based studies looking for maternal and child outcomes. Other issues are more difficult to address, for example the effect of health care and community-based services in the postpartum period. Also, innovative approaches to increase patients’ knowledge on reproduction issues and enhance self-management could be more emphasized. The motherhood decision support aid by Meade ^4^ is an example that utilization of computer-based technology can help to develop instruments that can be tested in randomized, controlled trials. Table 2 would have gained by an additional column showing a proposal for research design and methods.

 The working group reached a consensus that research focusing on improving shared decision-making in healthcare, high quality conversations during the pre-conception stage, evidence-based information on medication use during pregnancy and breastfeeding, and more personalised approaches to care had a high priority. Obviously the already existing specialized pregnancy/family planning clinics could initiate the research that is proposed.

 One major limitation of the study mentioned by the authors is it generalisability. Patient-centred care is promoted in several countries but is definitely lacking in many others where cultural and religious attitudes prevent predominantly women from shared-decision making. 

 By ranking research issues the paper raises awareness related to family planning, pregnancy, and early parenting in a group of chronic diseases that preferentially affect women. The paper will truly stimulate research and result in more standardized interventions that effectively support female patients on their way to motherhood. The key to improve the patient situation is to communicate the knowledge acquired through research, to disseminate it and to make it accessible in every-day language to the patient.

I have read this submission. I believe that I have an appropriate level of expertise to confirm that it is of an acceptable scientific standard.

## References

[1] AndreoliLBertsiasGKAgmon-LevinNBrownSCerveraRCostedoat-ChalumeauNDoriaAFischer-BetzRForgerFMoraes-FontesMFKhamashtaMKingJLojaconoAMarchioriFMeroniPLMoscaMMottaMOstensenMPamfilCRaioLSchneiderMSvenungssonETektonidouMYavuzSBoumpasDTincaniA: EULAR recommendations for women's health and the management of family planning, assisted reproduction, pregnancy and menopause in patients with systemic lupus erythematosus and/or antiphospholipid syndrome. *Ann Rheum Dis*.2017;76(3) : 10.1136/annrheumdis-2016-209770 476-485 10.1136/annrheumdis-2016-209770 27457513PMC5446003

[2] OstensenMRugelsjoenA: Problem areas of the rheumatic mother. *Am J Reprod Immunol*.28(3-4) :254-5 128589510.1111/j.1600-0897.1992.tb00808.x

[3] ChakravartyEClowseMEPushparajahDSMertensSGordonC: Family planning and pregnancy issues for women with systemic inflammatory diseases: patient and physician perspectives. *BMJ Open*.2014;4(2) : 10.1136/bmjopen-2013-004081 e004081 10.1136/bmjopen-2013-004081 24500612PMC3918989

[4] MeadeTDowswellEManoliosNSharpeL: The motherhood choices decision aid for women with rheumatoid arthritis increases knowledge and reduces decisional conflict: a randomized controlled trial. *BMC Musculoskelet Disord*.2015;16: 10.1186/s12891-015-0713-0 260 10.1186/s12891-015-0713-0 26395873PMC4579637
